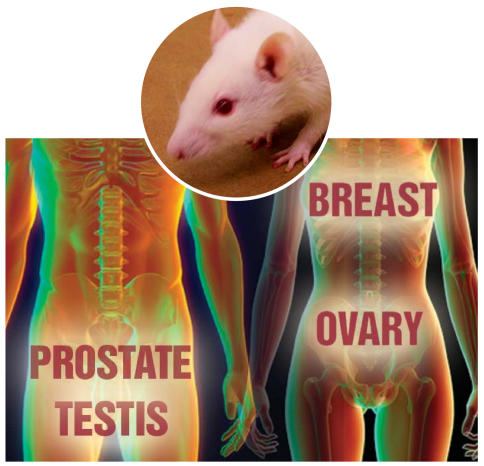# The Best Rodent for the Job: NTP Workshop Compares Models

**Published:** 2007-09

**Authors:** Julia R. Barrett

Researchers have long used rats and mice to understand the potential links between environmental exposures and incidences of breast, ovarian, testicular, and prostate tumors. Concern exists, however, that rodent models are not optimal for detecting carcinogens that act through the hormone system. To address this concern, the National Toxicology Program (NTP) organized a workshop in May 2006 to evaluate the utility of current two-year rodent bioassays to adequately evaluate these hormon ally mediated tumors and the relevance of the findings to humans **[*EHP* 115:1351–1356; Thayer and Foster]**.

The workshop, part of a series of events aimed at evaluating and refining the NTP’s testing program, included representatives from academia, industry, government, and nonprofit groups, as well as a panel of invited experts in endocrinology, cancer biology, reproductive toxicology, statistics, and other fields. Workshop participants were concerned that many hormonally mediated tumors, such as those in the testes and breasts, are initiated in fetal or early neonatal life, yet these periods of exposure are not covered in the NTP’s standard cancer bioassay. In response to this concern, the NTP has committed to routinely include perinatal exposures in these studies unless there is a specific justification not to do so.

Furthermore, some rat and mouse strains used in testing either do not develop certain tumors or have a high incidence of spontaneous tumors. For example, the F344/N rat typically used by the NTP has high background incidences of testicular Leydig cell tumors and mononuclear cell leukemia, along with unresolved issues about declining fertility, sporadic seizures, and chylothorax (accumulation of lymphatic fluid in the pleural cavity, which can result from lymphoma). By press time, in response to this and other workshop findings, the NTP had selected the Wistar Han as its standard rat strain cancer and noncancer end points, although other strains will be used when appropriate.

Although rodent models are considered to have certain deficiencies and can be improved, they are considered valuable nonetheless. Rodent models for prostate and ovarian tumors are the most problematic for understanding human disease because of significant interspecies differences in anatomy and tumor prevalence. Participants in the workshop recommended using alternative models, such as genetically engineered models and *in vitro* systems, to address some of these deficiencies. They also recommended more in-depth investigation of how noncancerous changes observed in rodents might be relevant to human diseases.

## Figures and Tables

**Figure:**